# 3D Cultures and Organoids in Cancer Research

**DOI:** 10.3390/cancers18213517

**Published:** 2026-11-02

**Authors:** César López-Camarillo, Karla Rubio

**Affiliations:** 1 Posgrado en Ciencias Genómicas, Universidad Autónoma de la Ciudad de México, Mexico City 03104, Mexico; 2 Laboratorio Internacional EPIGEN, Dirección de Innovación y Transferencia de Conocimiento, Benemérita Universidad Autónoma de Puebla, Puebla 72570, Mexico; epigen.concytep@puebla.gob.mx

Three-dimensional (3D) cell cultures resemble tumor spatial architecture in vitro, offering a groundbreaking approach to uncovering complex cancer biology and identifying vulnerabilities. 3D cultures better recapitulate the molecular features of tumors, including cancer cell behavior and oxygen and nutrient gradients. Moreover, 3D cultures can recapitulate tumor transcriptional and epigenetic programs, including oncogenic signaling pathways and aberrant cellular and molecular processes [1]. Recent advances in organoid technologies have allowed the building of 3D cellular cultures from patient-derived tumors [2]. The 3D cultures containing stroma and extracellular matrix components may provide insights into the genetic programs underlying cancer development and progression. Also, they can give valuable information about the influence of the 3D microenvironment on the reprogramming of non-coding RNAs and mRNA gene expression patterns. The main advantages of 3D cultures include the identification of key genes and proteins potentially translated into novel therapeutic strategies in cancer.

This Special Issue provides original research and review articles covering recent advances in understanding the role of innovative 3D cell cultures and patient-derived organoids in elucidating the mechanisms of cancer development and progression, drug screening, and applications in personalized medicine. To encourage readers to access the collection, here we introduce the representative contributions made by the authors.

The review by Flores-García and coworkers integrates the available evidence regarding the impact of 3D systems on the epigenetic and transcriptional reprogramming of breast cancer [3]. Based on a systematic search of 133 studies, the authors analyzed changes in DNA methylation, histone modifications, chromatin organization, and non-coding RNA expression, comparing 2D and 3D cultures, as well as various 3D platforms using matrices that differ in composition, stiffness, porosity, and concentration. The evidence suggests that 3D architecture serves not merely as a physical scaffold but also as a regulatory stimulus capable of altering cellular activity and epigenetic programs. One proposed mechanism involves the activation of mechanotransduction pathways associated with proteins such as FAK, ILK, Rho, and YAP. These pathways can modify chromatin accessibility and gene expression, thereby promoting changes in tumor cell plasticity. Regarding epigenetic modifications, DNA methylation patterns vary across 3D models. Some studies have identified specific alterations, such as hypermethylation of the miR-29c promoter and hypomethylation of HOX gene promoters. However, these modifications are not consistent across different matrices, as comparisons between Matrigel and alginate substrates revealed no significant differences in global DNA methylation patterns. In contrast, histone modifications exhibit more consistent patterns across different 3D systems, including Matrigel and polyHEMA formulations. Changes in histone acetylation and deacetylation can alter chromatin condensation and contribute to processes associated with tumor cell plasticity, metabolism, epithelial–mesenchymal transition, and therapeutic resistance. These epigenetic alterations also affect non-coding RNAs, including miRNAs, lncRNAs, and circRNAs, whose expression responds to the physical and biochemical characteristics of the microenvironment, including mechanotransduction-associated processes. Spheroids tend to exhibit changes associated with chemoresistance, metabolism, and proliferation, involving miRNAs such as miR-22, miR-29c, and miR-29b-3p. In contrast, cultures grown in Matrigel or Geltrex exhibit changes in miRNA expression associated with morphogenesis and WNT and MAPK signaling, accompanied by the overexpression of lncRNAs such as HOTAIR-N and AFAP1-AS1. Evidence regarding circRNAs remains limited, although some studies suggest that they participate in ceRNA-like regulatory networks during tumor transformation. Finally, the review highlights the lack of standardization across 3D models as a major limitation for comparing findings, largely due to differences in the physicochemical properties of matrices, batch-to-batch variability, and polymerization conditions. The authors propose that these variables should be reported and that different 3D systems should be systematically compared to distinguish epigenetic alterations associated with tumor biology from those arising from experimental conditions. In this context, HDAC inhibitors may represent potential therapeutic tools, given the reproducibility of histone modification changes observed across various 3D models.

Cancer organoids, or tumoroids, are patient-derived 3D cultures that mimic tumor biology. Organoids can be used as drug screening models capturing patient outcomes and clinical responses. In an original research paper, Ali and coworkers [4] describe the development of organoids from transurethral resection of the prostate (TUR-P), which traditionally has been hampered by a low success rate and the high cost of culture. The authors reported a novel protocol for optimal success and reduced fee of advanced prostate cancer organoids from TUR-P specimens. They established organoids in 62.07% of cases, using TUR-P clinical samples, and reported an optimized protocol, which improved the success rate and feasibility. Importantly, the authors showed that the organoids preserved histological and immunohistochemical markers, as well as genomic characteristics like the original tumors. The STR and ddPCR analyses confirmed conservation of alterations in BRCA1 and mutations in key genes such as TP53, FOXA1, and APC. In addition, exome sequencing showed an average similarity of 73.92% in mutations between tissues and organoids, maintaining mutations in TP53, PTEN, AR, BRCA1, and BRCA2 key genes.

A key finding was that the organoids predicted individual responses to treatments. The organoids showed sensitivity to docetaxel in line with the clinical response, reflected in the decrease in prostate-specific antigen (PSA). Remarkably, the therapy resistance in organoids corresponded to an increase in PSA in patients, achieving agreement in evaluated cases. Regarding prospects, the authors make the following remarks: (i) optimization of cultures for routine clinical use is required, and (ii) expanding the patient cohort is needed to validate predictive accuracy and improve success rates in organoid shunting. In conclusion, reported findings correlated with clinical outcomes, supporting their use in personalized treatment decisions.

The 3D organotypic culture models can also mimic epithelial stratification and differentiation, providing a useful system for studying cancer progression in vitro. In human papillomavirus (HPV)-associated oropharyngeal cancer, these models are particularly relevant because defined precursor lesions are lacking and early-stage samples are difficult to obtain. In an original research paper, Avila Tejeda and coworkers [5] developed a three-dimensional organotypic raft culture model to study the progression of human papillomavirus type 16 (HPV16)-associated oropharyngeal squamous cell carcinoma (OPSCC). Using HPV16-transfected primary human tonsil keratinocytes, the authors generated stratified epithelial tissues and examined changes associated with continuous passaging. The model simulated the progression of HPV16-transfected oral epithelium from precancerous states toward malignant phenotypes. With increasing passage number, the raft cultures showed progressive impairment of epithelial architecture and differentiation, together with decreased HPV16 virus titers and progressive viral genome integration. These changes were accompanied by altered expression of differentiation and carcinogenic markers, including increased K8 and p16 and decreased p53 and pRb. The authors also observed loss of p120 expression and increased COX5B expression during progression. Importantly, several of these expression patterns were consistent with those reported in HVP-associated OPSCC tissues. Overall, the study shows that the organotypic raft culture system can reproduce important features of HVP16-associated OPSCC progression in vitro. This model may therefore be useful for studying early and intermediate stages of disease and for identifying potential biomarkers and therapeutic targets.

Patient-derived tumor explants represent a valuable approach for functional precision oncology, allowing the evaluation of therapeutic responses directly from patient samples. This tool captures phenotypic and molecular response to therapy, including microenvironmental interactions and drug synergy, enabling the identification of tumor vulnerabilities. These approaches are particularly relevant for rare malignancies, where limited clinical samples and the absence of effective treatment strategies represent major challenges. In an original study, Schneider and coworkers investigated ex vivo tumor explant cultures to identify therapeutic vulnerabilities in fibrolamellar carcinoma (FLC), a rare primary liver malignancy that predominantly affects adolescents and young adults [6]. Using patient-derived tumor samples, the authors established an ex vivo functional precision oncology platform based on 3D explant cultures and evaluated responses to a panel of anticancer agents and drug combinations. This approach enabled the assessment of drug sensitivity directly from patient tumor specimens and provided functional response profiles for individual tumors. The authors analyzed 41 FLC samples and evaluated the activity of 24 therapeutic agents, identifying differential responses among tumors. Among the compounds tested, vorinostat, phenformin, and 6-diazo-5-oxo-L-norleucine showed higher activity in the ex vivo assays, while several drug combinations, including gemcitabine plus oxaliplatin and 5-fluorouracil plus interferon, demonstrated enhanced responses. In addition, metabolomic analyses revealed alterations associated with mitochondrial dysfunction and polyamine metabolism, providing additional information on the metabolic features associated with treatment response. Overall, this study demonstrates the utility of patient-derived tumor explant models as a functional platform for investigating therapeutic responses in rare cancers. Although further clinical validation is required, these findings support the use of ex vivo approaches to identify therapeutic vulnerabilities and evaluate potential personalized therapeutic approaches for fibrolamellar carcinoma.

In conclusion, organotypic 3D cell cultures and organoids are essential for understanding cancer biology and developing new therapeutic strategies for disease. The experimental discoveries, reviews, and opinions presented in this Special Issue may shape the future of oncology and inspire future research and general readers, scholars, and scientists.

## Data Availability

No new data were created or analyzed in this study.
